# Development of a novel strategy for fungal transformation based on a mutant locus conferring carboxin-resistance in *Magnaporthe oryzae*

**DOI:** 10.1186/s13568-016-0232-x

**Published:** 2016-08-24

**Authors:** Min Guo, Xiaolei Zhu, Hongxia Li, Leyong Tan, Yuemin Pan

**Affiliations:** 1 Department of Plant Pathology, College of Plant Protection, Anhui Agricultural University, Hefei, 230036 China; 2 Anhui Research Institute of Chemical Industry, Hefei, 230041 China

**Keywords:** Succinate dehydrogenase, Carboxin, Selectable marker, *Magnaporthe oryzae*

## Abstract

The accurate manipulation of genomic integration of mutant alleles or fluorescent fusion-protein constructs is necessary for understanding of pathogenic mechanism of *Magnaporthe oryzae*. Recently, this can be achieved by integrating of exogenous DNA randomly into genome of this pathogen, but ectopic integration may result in alteration of gene expression or gene disruption due to unpredictable position effects and/or disruption of protein-coding regions. In this study, we establish a novel strategy for locus-specific integration of exogenous DNA via carboxin-resistance reconstitution by a point-mutation (H245L) on succinate dehydrogenase subunit Mosdi1. Independent transformants derived from the same reconstitution construct showed consistent fluorescent signal and undiversified phenotypes, including hyphae growth, conidiation and pathogenicity, in *M. oryzae*. Meanwhile, 96 % of all transformants integrate correctly into the *Mosdi1* locus as a single copy. Furthermore, we provide a vector carrying yeast recombination cassette and thus allow assembly of multiple overlapping DNA fragments by yeast in vivo recombination for gene complementation and protein localization assay.

**Electronic supplementary material:**

The online version of this article (doi:10.1186/s13568-016-0232-x) contains supplementary material, which is available to authorized users.

## Introduction

Homologous recombination is regarded as a common phenomenon in nature, and now it is extensively used as an indispensable technology in genetic engineering. In fungal transformation, most plasmid DNA was integrated into the genome by non-homologous, ectopic recombination. Such random integration may result in alteration of gene expression or gene disruption by unpredictable position effects. However, such potential risks can be eliminated by targeted integration of genetic constructs into a defined locus without disrupting other regions of fungal genomic DNA (Weld et al. 2006). Till now, generally used loci for targeted integration in filamentous fungi include *pyrG* in *Aspergillus niger* (van Hartingsveldt et al. 1987), *ILV2* in *Magnaporthe oryzae* (Yang and Naqvi 2014) and *sdi1* in *Ustilago maydis* and *Zymoseptoria tritici* (Kilaru et al. 2015).

In filamentous fungi, succinate dehydrogenase (SDH), an essential enzyme involved in the respiratory chain, is composed by a FAD-containing flavoprotein subunit (SdhA), an iron–sulfur protein subunit (SdhB or sdi1), and two hydrophobic polypeptides (SdhC and SdhD) (Ulrich and Mathre 1972). Carboxin, a systemic fungicide against most fungi, is identified to inhibit the respiratory chain by affecting the enzyme activity of succinate dehydrogenase (SDH) (Skinner et al. 1998; Honda et al. 2000; Kilaru et al. 2009; Ando et al. 2009; Shima et al. 2009; Yin et al. 2011). Previous studies revealed that a mutation caused by a single amino-acid substitution (H245L) in the third cysteine-rich cluster of sdi1 subunit could confer resistance to carboxin in *U. maydis* (Broomfield and Hargreaves 1992; Keon et al. 1991), and thereafter was used as a valuable selection marker for homologous integration in several fungi (Skinner et al. 1998; Honda et al. 2000; Kilaru et al. 2009; Ando et al. 2009; Shima et al. 2009; Yin et al. 2011).

In *M. oryzae*, the plasmid pCB1532 and pYF11 were used for complementation and protein localization by randomly inserting targeted gene into genome of corresponding strains (Wang et al. 2013; Zhang et al. 2009). However, uncertain genes disruption may be occurred by random DNA integration during fungal transformation and the phenotype of deleted mutants could not be recovered as expected. Therefore, it encouraged us to develop a feasible strategy for locus-specific integration of foreign DNA in *M. oryzae*. In this study, we provide a vector pMoC-*eGFP* that was efficiently integrated into the *sdi1* locus as single copy by using carboxin as selectable agent. In spite of this, the introduction of foreign DNA at *Mosdi1* locus doesn’t alter the growth and virulence of *M. oryzae*. This tool promises to simplify the procedure for functional genomics studies in *M. oryzae*.

## Materials and methods

### Strains and culture conditions

The *M. oryzae* wild type strain Guy11 (ATCC201236) was used as recipient strain for fungal transformation. For vegetative growth, both Guy11 and its derivative mutants were cultured on solid CM (Complete medium) (Talbot et al. 1993) at 28 °C in darkness. For conidiation, *M. oryzae* strains were grown on RDC medium (Guo et al. 2011) and incubated at 28 °C for 10 days in darkness before exposed to continuous fluorescent light for another 3–5 days. Conidia were harvested by usage of glass rod to gently scrape the surface of colonies with 5 mL distilled water. The suspension was filtered through two layers of Miracloth (Calbiochem, SanDiego, USA) to remove mycelial debris, and then was measured using a haemocytometer. For conidium germination and appressorium formation, drops (20 μL) of conidial suspension (5 × 10^4^ spores mL^−1^) were treated as previously described (Guo et al. 2016) and observed by microscopic examination of at least 99 conidia per replicate at 24 hpi.

*Saccharomyces cerevisiae* FY834 (*MATα*; *his∆200*; *ura3*-*52*; *leu2∆1*; *lys2∆202*) was refreshed on YPD agar medium (per liter: 20 g glucose, 20 g peptone, and 10 g yeast extract) at 28 °C for 48 h and then used for preparing competent cells as described by Frozen-EZ Yeast Transformation II Kit (T2001, Zymo research, USA). Yeast transformants were selected on Sc-Ura medium (per liter: 1.7 g yeast nitrogen base, 5 g ammonium sulphate, 5 g casein hydrolysate, 20 mg adenine hemisulfate salt, 20 g glucose) and positive colony was validated by PCR amplification with primers GL559/GL565 (Additional file 1: Table S1). *Escherichia coli* strain DH5α was used for vector cloning and plasmid maintenance. *Agrobacterium tumefaciens* strain EHA105 (Hood et al. 1993) was used for *M. oryzae* transformation.

### Construction of a yeast-*Escherichia*-*Agrobacterium* shuttle vector pMoC

The plasmid pMoC is a vector built on the framework of binary vector pCAMBIA1300 (www.cambia.org). To construct pMoC vector, a 2.9-kb URA3-2micro2_origin fragment amplified from plasmid pYES2 (Invitrogen, USA) with primers GL729/GL730 (Additional file 1: Table S1) was digested with *Sac*II, and then inserted into *Sac*II site of pCAMBIA1300 by T4 DNA ligase (Sambrook et al. 1989).

### Construction of targeted ectopic integration vector pMoC-*eGFP*

Plasmid pMoC-*eGFP* was constructed using yeast strain FY834 following published procedures (Lu et al. 2014). To assemble DNA fragments using yeast in vivo homologous recombination system (Park et al. 2004), primers were designed with 30 bp DNA sequences homologous to upstream and downstream of the fragments to be cloned. To obtain pMoC-*eGFP* vector, a 9742 bp fragment of pMoC (digested by *Xho*I and *Eco*RI), 463 bp *Mosdi1* coding sequence (amplified by GL547/GL519 from *M. oryzae*), a point-mutated 476 bp fragments containing 98 bp of 3′ end *Mosdi1* gene and 378 bp downstream of the *Mosdi1* gene (amplified by GL520/GL562 from *M. oryzae*), 1505 bp *Morak1* promoter (amplified by GL580/GL689 from *M. oryzae*), 717 bp *eGFP* (amplified by GL564/GL565 from *M. oryzae*), a 635 bp *Morak1* terminator (amplified by GL583/GL581 from *M. oryzae*), and a 1026 bp fragment covering right flank of *Mosdi1* (amplified by GL568/GL556 from *M. oryzae*) were mixed together and transformed into the FY834 competent cells by PEG/LiAc-mediated transformation. The yeast plasmid DNA, purified using yeast plasmid DNA extraction kit (D1160, Solarbio, Beijing, China), was firstly transformed into *E. coli* competent cells, and then isolated for DNA sequencing. The sequence verified plasmid pMoC-*eGFP* was transformed into EHA105 strain by the established method (Holsters et al. 1978). Primers used are listed in Additional file 1: Table S1.

### *Agrobacterium tumefaciens*-mediated transformation of *M. oryzae*

*Agrobacterium tumefaciens*-mediated transformation of *M. oryzae* was following the published procedures (Chen et al. 2011). The modified *A. tumefaciens* EHA105 strain, which contains the plasmid pMoC-*eGFP*, was freshly cultured, and then mixed with an equal volume of *M. oryzae* conidial suspensions (1 × 10^6^ spores mL^−1^), and subsequently 200 µL of the mixed cultures were spreading on cellulose nitrate membranes (CNM, 11406-47-ACN, Sartorius Biotech, Goettingen, Germany) that is placed over the co-cultivation medium and grown at 22 °C for 48 h. The CNM were transferred to half CM plates (Chen et al. 2011) with 100 µg mL^−1^ cefotaxime (C8240, Solarbio, Beijing, China), 100 µg mL^−1^ timentin (T8660, Solarbio, Beijing, China) and 50 µg mL^−1^ carboxin (45371, Sigma-Aldrich, Germany) and then incubated at 28 °C in darkness until the colonies appear. The individual colonies were transferred to CM agar plates with antibiotics described above and grown at 28 °C for 3–4 days.

### Characterization of transformants by PCR, Southern blotting

Genomic PCR amplification was used to validate the specific integration vector pMoC-*eGFP* into the *Mosdi1* locus of *M. oryzae* by a set of primers GL728/GL565. DNA hybridization probes were amplified with primers GL726/GL727 and then labeled with digoxigenin-11-dUTP using DIG-High prime according to the instructions (11745832910, Roche, Shanghai, China). For Southern blot, genomic DNA of the transformants was digested by *Bam*HI and hybridized with the DIG-labeled probes using standard protocols (Sambrook et al. 1989). Primers used are listed in Additional file 1: Table S1.

### Microscopy

A manual inverted microscope (*Ti*-S; Nikon, Tokyo, Japan) equipped with a Super Plan Fluor ELWD ADM 20/0.45 was used to observe the eGFP expression in mycelia, conidia, germ tube, appressorium and invasive hyphae. eGFP was exited using a HG fiber illuminator with filter cube (Excitation Filter EX450-490, Dichroic Mirror DM505, Barrier Filter BA520, Tokyo, Japan) and images were acquired using a high-resolution color camera head DS-Ri2 (Nikon, Tokyo, Japan). All parts of the system were under the control of the NIS-elements F (Nikon, Tokyo, Japan).

### Plant infection assays

14-day-old susceptible rice seedlings (*Oryza sativa* cv Co39) and/or 7-day-old barley leaves was used for plant infection assay. The rice seedlings infection assay was carried out following the established procedures (Guo et al. 2016). The disease symptom was assessed at 5 days post inoculation (dpi). The barley leaves infection was performed as previously described (Wang et al. 2013). The disease severity was assessed at 5 dpi. Plant penetration assays were carried out using 7-day-old barley leaves following procedures by Guo et al. (2016). Invasive growth inside plant cells was examined at 48 h post inoculation (hpi).

### Multiple sequence alignment and phylogenetic analysis

Sdi1 protein sequences were obtained from NCBI database (www.ncbi.nlm.nlh.gov) using BLAST algorithm (McGinnis and Madden 2004). Sequence alignments were performed using the ClustalW program (Thompson et al. 1994).

## Results

### Point mutation of Mosdi1 conferring resistance to carboxin

**Fig. 1:**
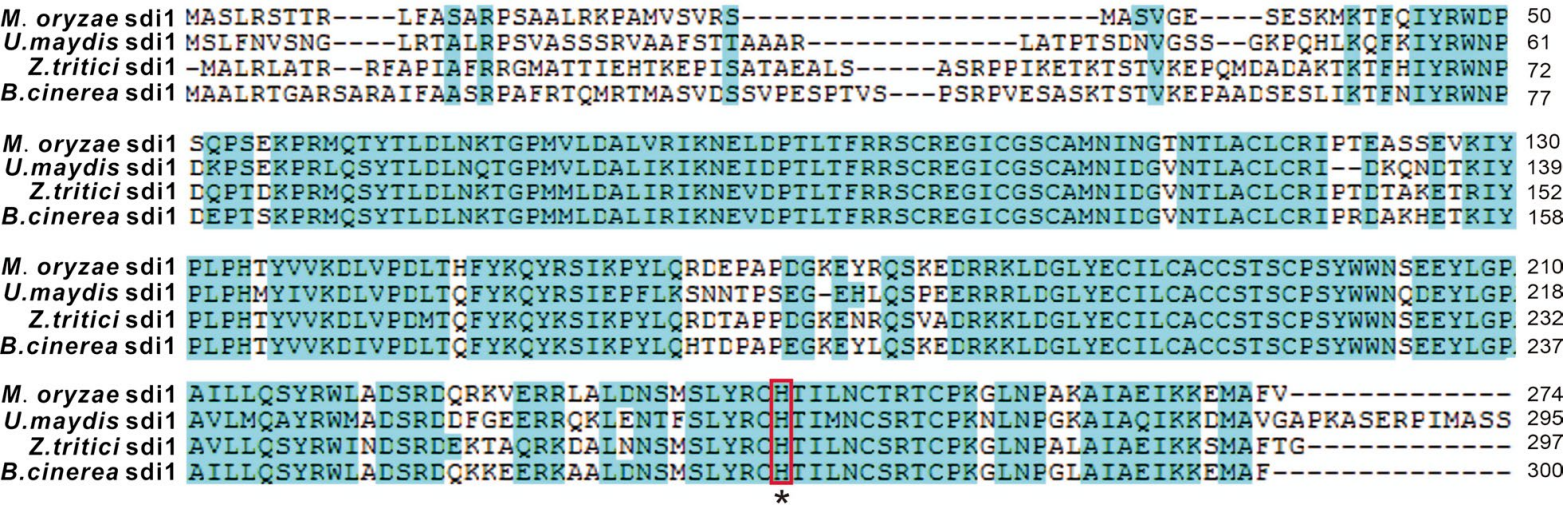
Comparison of the amino acid sequences of succinate dehydrogenase subunit from *M. oryzae* (XP_003718958), *U. maydis* (Accession No., XP_011386878), *Z. tritici* (Accession No., XP_003850753) and *B. cinerea* (Accession No., XP_003850753). Identical amino acids are highlighted by *blue background*. Note that the critical histidine at position 245 in *M. oryzae* is conserved in all the organisms (indicated in *red rectangle*)

**Fig. 2:**
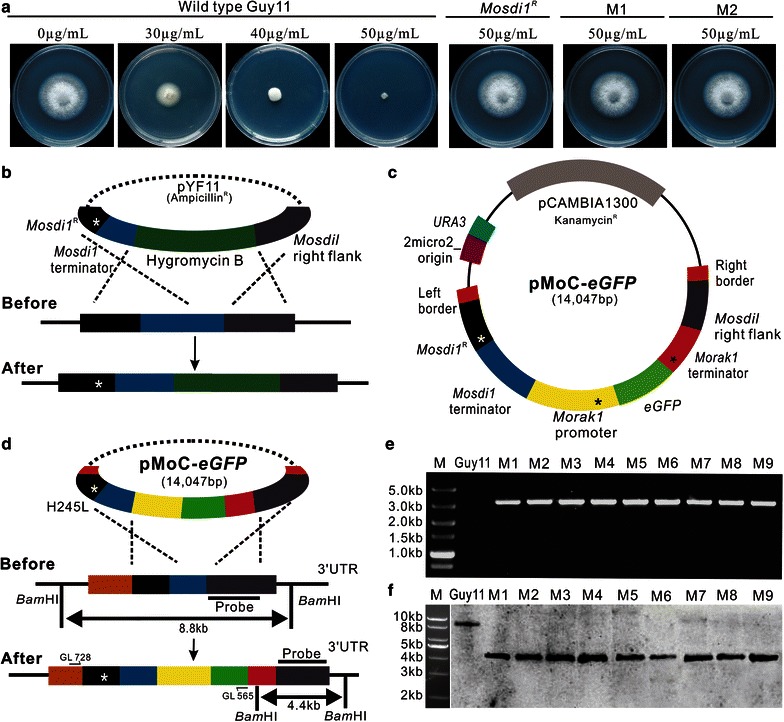
Construction of a vector for targeted integration into the *Mosdi1* locus in *M. oryzae.* **a** The effect of carboxin against mycelial growth of *M. oryzae* strains on complete media. *M. oryzae* wild type Guy11 and its derivative mutants were cultured for 3 days on complete media supplemented with various concentrations of carboxin. *Mosdi1* ^*R*^ is a *Mosdi1* point mutation strain; M1, M2 are two strains integrated with vector pMoC-*eGFP* (*Mosdi1* ^*ReGFP*^). **b** Strategy for generating *Mosdi1* point mutation mutant. **c** Schematic drawing showing the organization of vector pMoC-*eGFP*. The fluorescent protein eGFP is expressed under the *M. oryzae Morak1* promoter. *URA3* and 2 micro2_origin cassettes enable yeast recombination-based cloning in *S. cerevisiae*. After integration into the *Mosdi1* locus, a point mutation in the succinate dehydrogenase encoding gene *Mosdi1*, which resulted in substitution of histidine to leucine (H245L), could confer carboxin resistance for the transformants. Note that fragments are not drawn to scale. For more accurate information on fragment sizes see main text. **d** Image illustrates the integration of pMoC-*eGFP* into the native *Mosdi1* locus of *M. oryzae*. In this integration, the carboxin-resistant *Mosdi1* ^*R*^ allele and cytoplasmic eGFP were simultaneously inserted into the genome of *M. oryzae.* **e** The *Mosdi1* ^*ReGFP*^ mutants (M1–M9) were validated by dialogistic PCR. Genomic DNA from *Mosdi1* ^*ReGFP*^ strains and wild-type strain Guy11 were extracted, respectively, and used for screening of the integration into *Mosdi1* locus by PCR. **f** The *Mosdi1* ^*ReGFP*^ mutants (M1–M9) were validated by southern blot. Single integration into the expected locus was found in all nine *Mosdi1* ^*ReGFP*^ mutants, and expected size bands were obtained in both *Mosdi1* ^*ReGFP*^ mutants and wild type strain Guy11. The size markers in the corresponding agarose gel are shown at the *left*

In *M. oryzae*, wild type Mosdi1 (GenBank accession, XP_003718958) encodes a 274 amino acid protein and shares 63, 72 and 77 % similarities to the corresponding sequences in *U. maydis* (GenBank accession, XP_756991), *Z. tritici* (GenBank accession, XP_003850753) and *B. cinerea* (GenBank accession, XP_001548350), respectively. Sequence alignments of proteins from different organisms revealed that the occurrence of histidine (245) is conserved in Mosdi1 (Fig. 1), suggesting a similar role in conferring resistance to fungicide carboxin (Skinner et al. 1998). To validate this hypothesis, the sensitivity of wild type strain Guy11 to carboxin was assayed, and the result showed that fungal mycelial growth was completely inhibited in CM amended with 50 μg mL^−1^ carboxin (Fig. 2a). We next generate a point mutation strain *Mosdi1* ^*R*^ using the strategy described in Fig. 2b, and found that, like many other fungi, amino acid substitution (H245L) in the Mosdi1 subunit (*Mosdi1* ^*R*^ mutants) could confer resistance to carboxin (Fig. 2a).

### Construction of a targeted integration vector based the *Mosdi*^*R*^ locus in *M. oryzae*

As *sdi1* ^*R*^ locus has been recommended for locus-specific integration of exogenous DNA in study of protein localization and gene complementation in filamentous phytopathogens (Kilaru et al. 2015, 2009; Shima et al. 2009), thus, a locus specific integration vector pMoC-*eGFP* based on mutated *Mosdi1* sequence was constructed. In this vector, an *eGFP* was controlled under the *M. oryzae Morak1* promoter and terminator sequences (Morak1 Accession No., XP_003710816). Meanwhile, the *Mosdi1* ^*R*^ left flank and right flank, which will facilitate targeted integration of foreign DNA into wild type *Mosdi1* locus, was introduced in the vector. In *Mosdi1* ^*R*^ left flank, a point mutation, which resulted in an amino-acid substitution (H245L), is made to acquire carboxin resistance (Fig. 2c). In fungal transformation, the integration by homologous recombination will mutate endogenous *Mosdi1* gene (Fig. 2d, indicated by white asterisk) and thus only those transformants undergoing recombination will survive on carboxin containing medium (Fig. 2a; M1 and M2). Besides, a yeast recombination cassette was introduced into the vector, which allowed multiple overlapping DNA fragments to be assembled in one step by yeast in vivo homologous recombination system. Furthermore, a *Hin*dIII restriction site (Fig. 2c, indicated by black asterisk) in both *Morak1* promoter and terminator sequences will facilitate exchange of targeted genes, fluorescent markers and tags in the pMoC-*eGFP* plasmid by yeast in vivo homologous recombination system to obtain new vectors without change of the *Mosdi1* ^*R*^ sequence.

### Generation of *Mosdi1*-based eGFP-labeled strains in *M. oryzae*

To confirm the efficiency of targeted integration of pMoC-*eGFP* into endogenous *Mosdi1* locus of *M. oryzae*, we transform the vector into Guy11 to generate *Mosdi1* ^*R*^ mutants expressing eGFP (*Mosdi1* ^*ReGFP*^) (Fig. 2a; M1 and M2 indicated strains of *Mosdi1* ^*ReGF*^). To validate targeted integration of pMoC-*eGFP* into endogenous *Mosdi1* locus, genomic DNA extracted from nine randomly selected *Mosdi1* ^*ReGFP*^ mutants was used for PCR screening. A 3.2 kb DNA fragment was obtained (amplified with GL728/GL565) in nine *Mosdi1* ^*ReGFP*^ mutants but not in Guy11 (Fig. 2d, e). Moreover, Southern blot showed that a single 4.4 kb band with expected size was identified in all nine *Mosdi1* ^*ReGFP*^ mutants while an 8.8 kb band was found in wild type (Fig. 2d, f). To confirm the advantage of this vector, we further used this targeted integration method to construct *Mopmt2* (Guo et al. 2016) and *Morak1* (unpublished) complemental strains and found that 96 % of the transformants has a single copy of integration into *Mosdi1* locus while the remaining 4 % of them has an additional integration event, with only native *Mosdi1* being replaced by carboxin-resistant allele *Mosdi1* ^*R*^ (in total 56 transformants), indicating that *Mosdi1* locus is an efficient target for ectopic integration of genetic constructs in *M. oryzae*.

### Phenotypes of eGFP-labeled transformants of *M. oryzae*

**Fig. 3:**
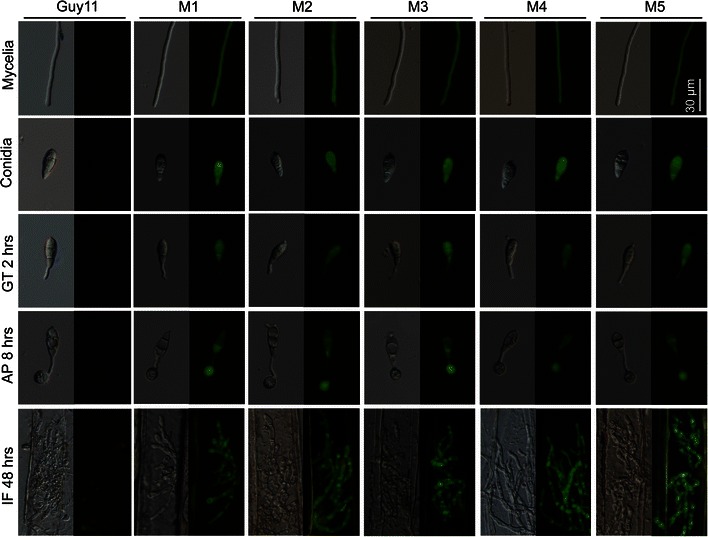
Cytoplasmic eGFP expression at different developmental stages in *Mosdi1* ^*ReGFP*^ mutants. All the samples for microscopic examination were treated as described in the main text and observed by a manual inverted fluorescent microscope (*Ti*-S, Nikon, Tokyo, Japan). Images from all independent transformants showed equivalent fluorescent signal in hyphae, conidia, germ tube, appressorium and invasive hyphae after integration of pMoC-*eGFP* into the *Mosdi1* locus. *Scale bar* 30 μm

To figure out whether pMoC-*eGFP* could integrate into the genome and express eGFP in the cytoplasm of *M. oryzae*, five randomly selected *Mosdi1* ^*ReGFP*^ mutants (M1–M5) were examined at different stages. They showed cytoplasmic eGFP expression in hyphae, conidia, germ tubes and appressorium, and displayed similar intensity of fluorescent signal in above cells. In addition, the eGFP in invasive hyphae also displayed equivalent fluorescent signal in all five *Mosdi1* ^*ReGFP*^ mutants (Fig. 3), indicating that the pMoC-*eGFP* was integrated into expected locus, with stable expression of eGFP in cytoplasm of *M. oryzae*.

**Fig. 4:**
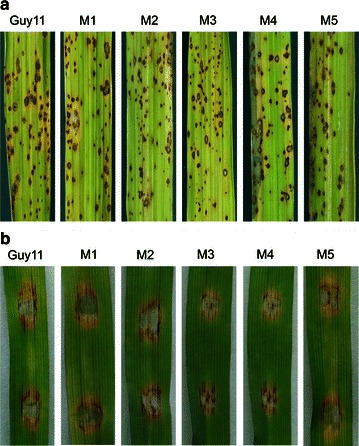
Pathogenic assay for *Mosdi1* ^*ReGFP*^ mutants. The pathogenicity was tested for both wild type Guy11 and *Mosdi1* ^*ReGFP*^ mutants, and no significant differences were identified among the strains. **a** Disease symptoms caused by strains on 14-day-old rice seedlings. **b** Disease symptoms caused by strains on 7-day-old barley leaves

To investigate the impact of integration of pMoC-*eGFP* in *Mosdi1* locus of *M. oryzae*, mycelial growth of *Mosdi1* ^*ReGFP*^ mutants and Guy11 were compared on CM, MM, V8 (4 % V8 juice), OM (50 g of oatmeal per liter) and RDC media, and no significant differences were observed (Table 1). In addition, phenotypes like conidiation, conidium size, conidial germination and appressorium formation were also similar to each other (Table 1). The alteration of pathogenicity will result in failure of using this vector, therefore, the virulence of Guy11 and *Mosdi1* ^*ReGFP*^ mutants were compared on both barley and rice seedlings, but no obvious changes were obtained (Fig. 4a, b), indicating that integration of pMoC-*eGFP* vector in *M. oryzae* didn’t affect the fungal growth and virulence on host.

**Table 1:** Phenotypic summary of pMoC-*eGFP* targeted integration mutants

Strains	Conidiation (×10^5^ spores/cm^2^)	Conidium size (um)		Germination rate (%)	Appressorium formation (%)	Colony diameter (mm)				
		Length	Width			CM	MM	OM	RDC	V8
Guy11	1.03 ± 0.16^a^	23.14 ± 1.91^a^	8.83 ± 1.29^a^	95.43 ± 1.13^a^	98.04 ± 1.96^a^	38.23 ± 0.25^a^	32.30 ± 0.70^a^	33.63 ± 0.57^a^	33.93 ± 0.31^a^	35.03 ± 0.15^a^
M1	1.08 ± 0.08^a^	24.43 ± 2.08^a^	8.67 ± 1.38^a^	95.43 ± 2.99^a^	99.35 ± 1.13^a^	38.20 ± 0.30^a^	31.77 ± 0.25^a^	33.77 ± 0.25^a^	33.77 ± 0.25^a^	34.83 ± 0.15^a^
M2	1.03 ± 0.21^a^	22.61 ± 1.69^a^	8.41 ± 1.15^a^	94.12 ± 1.96^a^	97.39 ± 1.13^a^	37.83 ± 0.29^a^	31.93 ± 0.12^a^	33.83 ± 0.25^a^	33.63 ± 0.45^a^	34.70 ± 0.26^a^
M3	0.94 ± 0.08^a^	23.10 ± 1.79^a^	8.99 ± 1.25^a^	93.47 ± 4.08^a^	98.69 ± 1.13^a^	38.00 ± 0.20^a^	32.10 ± 0.36^a^	33.90 ± 0.36^a^	34.07 ± 0.40^a^	34.83 ± 0.15^a^
M4	1.03 ± 0.08^a^	23.10 ± 1.79^a^	8.88 ± 1.09^a^	94.12 ± 1.96^a^	97.39 ± 2. 39^a^	37.77 ± 0.25^a^	31.53 ± 0.50^a^	33.43 ± 0.21^a^	33.83 ± 0.35^a^	34.80 ± 0.20^a^
M5	0.98 ± 0.14^a^	21.79 ± 2.32^a^	8.66 ± 1.04^a^	94.77 ± 1.13^a^	98.69 ± 1.13^a^	37.90 ± 0.36^a^	32.00 ± 0.10^a^	33.77 ± 0.25^a^	33.83 ± 0.15^a^	34.77 ± 0.38^a^

Conidiation were measured by counting the number of conidia that harvested with 5 ml of sterilized water from 10-day-old RDC agar plates. The conidia sizes were measured with width and length from 99 conidia of each strain. Fungal mycelial growth rate was statistically analyzed by measuring the colony diameter of each strain on five different artificial media after 5 days after inoculation. For conidium germination and appressorium formation, drops (20 μL) of conidial suspension (5 × 10^4^ spores/mL) were inoculated onto a hydrophobic coverslip and placed under humid conditions at 28 °C. Conidia germination and appressoria formation were observed by microscopic examination of at least 99 conidia per replicate at each time point. All experiments were repeated three times, and representative results from one experiment are shown

* Lowercase letters indicate non-significant difference estimated by Duncan’s test (P < 0.05)

## Discussion

Up to date, various efforts have been made to improve the efficiency of gene targeting via homologous recombination (Jeong et al. 2007; Villalba et al. 2008), but few techniques have been developed for gene complementation and specific organelles labeling in *M. oryzae*. Recently, a new strategy has been constructed for genetic complementation and protein cellular localization by usage of vector pFGL889 in *M. oryzae* (Yang and Naqvi 2014). This plasmid has been built on the modified *Agrobacterium* T-DNA vector pFGL815 N using the *ILV2* ^*SUR*^ as the selectable marker (Yang and Naqvi 2014). However, by comparing with previously published vector pCeGFP in *Z. tritici* (Kilaru et al. 2015), which was built using yeast recombination-based cloning method, the disadvantages of using pFGL815 N as framework to construct new vectors were time consuming and limited to availability of suitable multiple cloning site. In this study, we draw on the experiences described in vector pFGL815 N and pCeGFP, and devised a novel strategy for site-specific integration of foreign DNA via carboxin resistance reconstitution by replacing the native *Mosdi1* with *Mosdi1* ^R^ allele. In vector pMoC-*eGFP*, an efficient yeast recombination-based cloning cassette was introduced to allow DNA fragments to be joined in various combinations and enables the rapid exchange of genes, promoters and dominant selectable marker cassettes without alteration of the coding sequences. Moreover, the mutated *Mosdi1* gene could serve as an additional selection marker in *M. oryzae* transformation, and is paramount for targeted insertions, gene complementation and protein cellular localization in *M. oryz*ae. In our previous transformation, the pMoC-*eGFP* showed a high efficiency (>96 %) of correct integrations into the genome of *M. oryzae*, and transformants harboring this vector displayed non-alteration of fungal virulence, suggesting the utility of *Mosdi1* locus for targeted integration is a useful method for genetic complementation analyses in *M. oryzae*.

## Additional file

**Additional file 1.** Primers used in this study.

## Abbreviations

CNM: cellulose nitrate membranes
eGFP: enhanced green fluorescent protein
CM: complete medium
MM: minimal medium
V8: V8 juice medium
OM: oatmeal agar medium
dpi: days post inoculation
hpi: hours post inoculation
*sdi1*^*R*^: carboxin resistant
