# Correction to: Development of a novel strategy for fungal transformation based on a mutant locus conferring carboxin-resistance in *Magnaporthe oryzae*

**DOI:** 10.1186/s13568-019-0814-5

**Published:** 2019-06-28

**Authors:** Min Guo, Xiaolei Zhu, Hongxia Li, Leyong Tan, Yuemin Pan

**Affiliations:** 1 Department of Plant Pathology, College of Plant Protection, Anhui Agricultural University, Hefei, 230036 China; 2 Anhui Research Institute of Chemical Industry, Hefei, 230041 China

## Correction to: AMB Expr (2016) 6:57 10.1186/s13568-016-0232-x

Following publication of the original article (Guo et al. 2016), the authors of Guo et al. (2016) would like to make a correction for a figure in their published article. An image in Fig. 4b (Lane 4, M3) were mistakenly used for strain M3. We checked the original images and replaced it with the correct Fig. 4b as showed below. We confirm that this change do not alter the findings of this work.

**Fig. 4:**
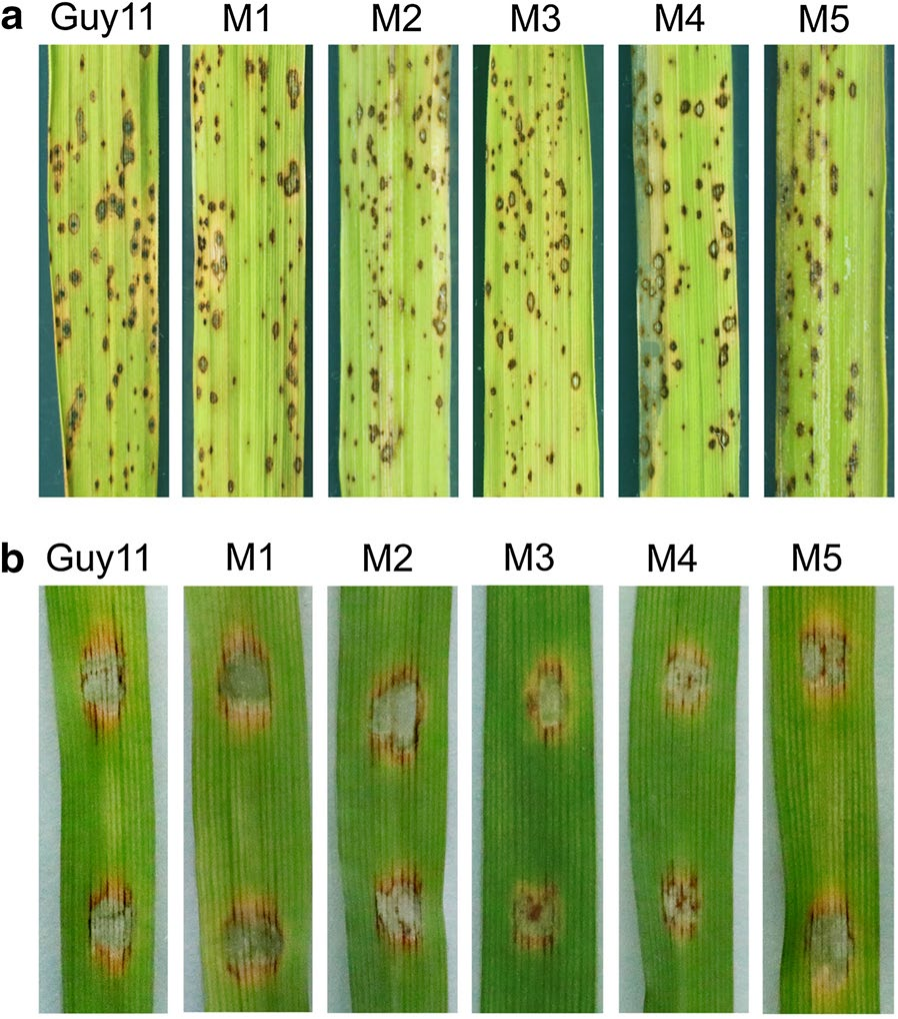
Pathogenic assay for *Mosdi1*^*ReGFP*^ mutants. The pathogenicity was tested for both wild type Guy11 and *Mosdi1*^*ReGFP*^ mutants, and no significant differences were identified among the strains. **a** Disease symptoms caused by strains on 14-day-old rice seedlings. **b** Disease symptoms caused by strains on 7-day-old barley leaves
