# Supplementary material for: Development of a novel strategy for fungal transformation based on a mutant locus conferring carboxin-resistance in Magnaporthe oryzae
Source: AMB Express. 2016 Aug 24;6:57. doi: 10.1186/s13568-016-0232-x (PMC4996803; doi:10.1186/s13568-016-0232-x)
Supplement: Supplementary file 1 — Additional file 1. Primers used in this study. [file 13568_2016_232_MOESM1_ESM.doc]

**Table S1.** Primers used in this study.

| Primer name | Primer sequences (5´-3´) | Remark |
| --- | --- | --- |
| GL547 | *TGGCAGGATATATTGTGGTGTAAACAAATT*GAGGCTTCTTCCGAAGTCAAG | *sdi1* left flank |
| GL519 | CAGTTAAGAATGGTGAGGCAAC |
| GL520 | GTACCGTTGCCTCACCATTC | 98 bp of 3’ end *Mosdi1* gene and 378bp downstream of the *Mosdi1* gene |
| GL562 | GTGACCTAGAAACCGACGAAG |
| GL580 | *CTACCCAAACTTCGTCGGTTTCTAGGTCAC*CCAACAGACTACGAGATAAG | *Morak1* promoter |
| GL689 | *GGTGAACAGCTCCTCGCCCTTGCTCACCAT*ATTTGCAAGTTTGCTGTGTG |
| GL564 | ATGGTGAGCAAGGGCGAGGAGC | eGFP |
| GL565 | TTACTTGTACAGCTCGTCCATG |
| GL583 | *ACTCTCGGCATGGACGAGCTGTACAAGTAA*ATGACCTCCGGTTGATGGGT | *Morak1* terminator |
| GL581 | *CCATGCATAAATGGGTGGAGATGCGTTCAC*AGCGTAATCAATCGCCTCCA |
| GL568 | GTGAACGCATCTCCACCCA | right flank of *Mosdi1* |
| GL556 | *TAAACGCTCTTTTCTCTTAGGTTTACCCGC*GTCGTAGCCAAGCTGAAGAAGGCGG |
| GL559 | TGATGGGCTGCCTGTATCGAGT | Yeast colony PCR |
| GL565 | TTACTTGTACAGCTCGTCCATG |
| GL729 | CCGCGGGGAACAACACTCAACCCTA | URA3-2micro2_origin fragment amplification |
| GL730 | CCGCGGTTCGATGTAACCCACTCG |
| GL726 | GCATCTCCACCCATTTATGC | DNA hybridization probes amplification |
| GL727 | GTCGTAGCCAAGCTGAAGAAG |
| GL728 | CTATAAGCCATGTACAGGTGC | Genomic PCR amplification |
| GL565 | TTACTTGTACAGCTCGTCCATG |

^a^ Italics indicate part of the primer that is complementary with another DNA fragment, to be ligated by homologous recombination in *S. cerevisiae*. Underlined letters indicated enzyme digestion site of *Sac*II.
